# Effect of Ovarian Cyclic Status on *In Vitro* Embryo Production in Cattle

**Published:** 2011-02-20

**Authors:** Akbar Pirestani, Sayyed Morteza Hosseini, Mahdi Hajian, Mohsen Forouzanfar, Fariba Moulavi, Parvaneh Abedi, Hamid Gourabi, Abdolhossein Shahverdi, Ahmad Vosough Taqi Dizaj, Mohammad Hossein Nasr Esfahani

**Affiliations:** 1 Department of Animal Science, Faculty of Agriculture, Islamic Azad University, Khorasgan Branch, Isfahan, Iran; 2 Department of Reproduction and Development, Reproductive Biomedicine Center, Royan Institute for Animal Biotechnology, ACECR, Isfahan, Iran; 3 Department of Animal Science, Marvdasht Branch, Islamic Azad University, Marvdasht, Iran; 4 Department of Genetics, Royan Institute for Reproductive Biomedicine, ACECR, Tehran, Iran; 5 Department of Embryology, Royan Institute for Reproductive Biomedicine, ACECR, Tehran, Iran; 6 Department of Reproductive Imaging, Royan Institute for Reproductive Biomedicine, ACECR, Tehran, Iran; 7 Department of Andrology, Royan Institute for Reproductive Biomedicine, ACECR, Tehran, Iran

**Keywords:** Embryo, Estrous Cycle, Cattle, Ovary

## Abstract

**Background:**

The relationship between cyclic status of cattle ovaries on *in vitro* embryo development up to the blastocyst stage was investigated.

**Materials and Methods:**

Cattle ovaries were collected immediately after slaughter and divided into three categories based on their cyclic status, which included: 1. the presence of a large follicle (LF), 2. the presence of a corpus luteum (CL) and 3. ovaries without LF or CL (WLCF). Oocytes of these ovaries were obtained and used for *in vitro* maturation and fertilization. Presumptive zygotes were then cultured up to the blastocyst stage in synthetic oviductal fluid culture medium.

**Results:**

There were no significant differences between cleavage rates of the three groups. The rate of embryos in the compact morula stage for the CL group was 48.2% which was significantly higher than the related rate of the LF group (36.6%), but non-significantly higher than that of the ST group (45.7%). The highest blastocyst rate belonged to the CL group (54.6%) which was significantly greater than the WLCF group (32.9%) and non-significantly higher than the LF group (52.4%). There was no significant difference in blastocyst rates in the CL and LF groups.

**Conclusion:**

Preselection of oocyte donor ovaries containing a CL or LF can be used as a feasible and non- invasive criterion to obtain the most competent oocytes capable of development to the blastocyst stage.

## Introduction

Since the birth of the first calves derived from *in vitro* fertilization of *in vitro* matured oocytes in 1990 (1), abattoir-derived ovaries have been the most important source of oocytes for embryologists working with domestic animals (2). Moreover, the majority of the several thousand cattle, sheep and pigs cloned in recent years (3, 4) have been produced by the transfer of their original somatic cell nuclei into enucleated oocytes which have been retrieved from abattoir ovaries. Therefore, it can be stated that without free access to an abundant source of abattoir-derived ovaries, the majority of the embryology laboratories could not and can not continue their research.

Ovaries collected from a slaughterhouse are from cattle at different stages of the estrous cycle, and hence, obtained ovaries vary regarding the presence of large follicle(s) (LF) or a functional corpus luteum (CL) (5). In this situation, the developmental competences of oocytes recovered from these ovaries may not be similar.

During the follicular wave of an ovary, a pool of primary oocytes is recruited to initiate growth and development in each wave, although just one wave in each estrous cycle progresses toward the ovulation of a fully competent secondary oocyte (6, 7). This dominant follicle controls growth of the other follicles through secretion of a variety of hormones such as oestradiol, inhibin, activin and follistatin, and other secretory products such as growth and inhibiting factors. These molecules may act locally, systematically or both locally and systemically (6, 7).

Upon ovulation, the ovulated follicle forms a CL through a cell differentiation process called luteinization. CL strictly controls ovarian functions through the secretion of estrogen and progesterone, as well as through secretion of locally acting molecules which regulate follicular waves (8, 9). Vassena et al. (10) have reported that a positive relationship exists between the regression of the early follicle and the competence of the retrieved oocyte. In another study, in sheep, Gonzalez-Bulnesa et al. investigated the effect of CL presence on in vivo and *in vitro* sheep embryo production (2). They found that the presence of a CL had no effect on either follicular numbers and sizes or on the number, morphology and ability of the retrieved oocytes on the resumption of meiosis after *in vitro* maturation. Importantly, oocytes retrieved from ovaries with CL had significantly higher capability to cleave, develop to the blastocyst stage and hatch after vitrification. In cattle, however, it is not clear if the presence of CL and LF on bovine ovaries at culling influences oocyte maturation and subsequent developmental competence up to the blastocyst stage. Moreover, the exact relationship between LF and oocytes recovered from those ovaries has not been understood. Therefore in the present study, we investigated the effect of ovary status of high genetic merit cows at culling on *in vitro* developmental competence up to the blastocyst stage.

## Materials and Methods

### Chemicals and media

Unless specified, all chemicals and media were obtained from Sigma Chemical Co. (St. Louis, MO, USA) and Gibco (Grand Island, NY, USA), respectively.

### Vero monolayer preparation

Frozen cryovials of an established Vero cell line were obtained from Royan Institute (www.royaninstitute. org) and used for this study as described elsewhere (11). In brief, each cryovial was quickly thawed at 37°C and the contents of the cryovial were diluted (1:4) with DMEM plus 10% fetal calf serum the sentence is correct (FCS) and centrifuged at 1500 rpm for 15 min. Washed and centrifuged Vero cells at a concentration of 1×10^6^/ml were cultured in 3 cm2 culture dishes which contained DMEM medium supplemented with 10% FCS at a temperature of 38.5°C and 5% CO2 in humidified air. The confluent dishes were trypsinised (0.25% trypsin) and detached, and single cells were either sub-cultured (to sustain the reserve cell source) or used for monolayer preparation. For the latter purpose, cells were diluted in the appropriate amount of SOF plus 10% FCS to a final concentration of 2×10^5^/ml.

### Ovary preparation and categorizing

**Fig 1:**
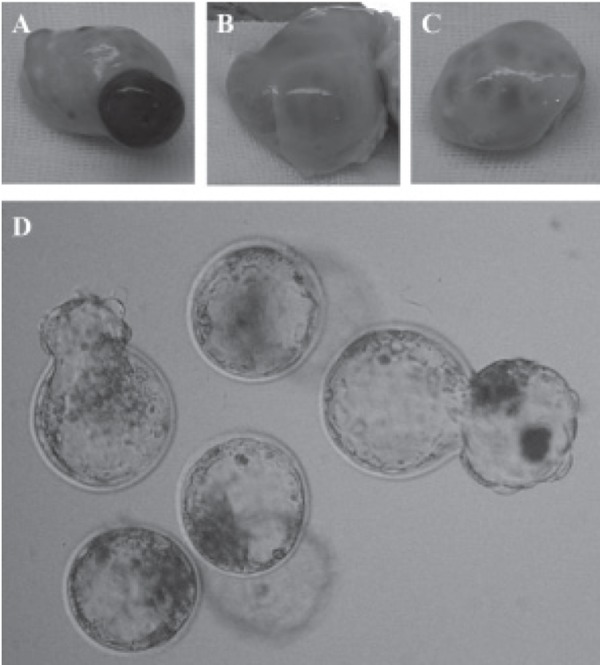
A-C: Morphology of ovaries used for obtaining oocytes. A. Ovary containing a large corpus luteum, B. ovary with a large follicle, C. ovary with no large corpus luteum or follicle. D. *in vitro* developed blastocysts.

Ovaries of each cow were collected immediately after slaughter, put in separate bags containing warm (30-35°C) normal saline plus antibiotic and transported to the laboratory within 2-3 hours. Collected ovaries were divided into three categories based on their cyclic status: 1. ovaries with LF (LF group), 2. ovary having at least a CL (CL group) and 3. ovaries without LF and CL (WLCF group). Figure 1 shows the ovaries in each category.

### *In vitro* oocyte maturation

Immature cumulus oocyte complexes (COCs) were obtained from ovaries by the slicing method (12). The procedures of *in vitro* maturation were carried out as described previously (13). In brief, the suspension which resulted from slicing was released into 12 cm petri dishes and with a stereomicroscope, COCs which contained homogenous cytoplasm and at least three surrounding cumulus cells were collected. Selected COCs were cultured in the presence of an established monolayer of Vero cells (approximately 1×10^5^ cells/ml) in 100μl droplets of maturation medium (10 COCs/droplet), covered with mineral oil at 39.0ºC, 5% CO2 and humidified air for 22-24 hours. Maturation medium was comprised of tissue culture medium 199 (TCM199) plus 10% FCS supplemented with 2.5 mM Na-pyruvate, 1mM L-glutamine, 100 IU/ ml penicillin, 100 μg/ml streptomycin, 10 μg/ml FSH, 10 μg/ml LH, 1μg/ml estradiol-17β and 0.1 mM cysteamine.

### *In vitro* fertilization and embryo culture

The procedures of IVF and IVC were carried out as described previously (13). In brief, 20-22 hours post-maturation all matured COCs with expanded and modified cumulus cells were selected and washed three times in fertilization medium. In each group at least ten COCs were allocated into 50 μl droplets of fertilization medium. Throughout this study, frozen semen straws of two bulls with proven fertility were used. For each treatment at least two 0.25 ml straws were quickly thawed at 37°C and the contents of the straws were centrifuged at 1200 rpm for 10 minutes. For *in vitro* fertilization, washed and prepared spermatozoa were loaded into the fertilization droplets at a final concentration of 1×10^6^/ml and co-incubated with matured COCs for 18-20 hours at 39°C in 5% CO2. The presumptive zygotes were denuded from the cumulus cells and washed twice with *in vitro* culture medium. For in vitro culture, presumptive zygotes were allocated in micro droplets (50 μl) of B2 medium (INRAFrance) in the presence of a monolayer of Vero cells, covered with mineral oil, and incubated at 38.5°C and 5% CO2 in humidified air. Embryos were refreshed into new dishes each two days where, concurrently the numbers of embryos that cleaved and developed into 8-16 cell, morula and blastocyst stages were recorded.

### Statistical analysis

The analysis of variance (ANOVA) procedure was used for data analysis. The mean of treatments were compared with Duncan’s multiple range test at a 0.05% probability level. Chi-square test was also used for comparison between different treatments.

## Results

Table 1 indicates number of COCs obtained from ovaries in each category. This table also shows developmental competence of COCs in each category, after *in vitro* maturation and fertilization.

**Table 1:** Developmental competence of bovine cumulus oocyte complexes (COCs) extracted from ovaries containing at least one LF, one CL and no LF or CL, after *in vitro* maturation and fertilization.


Ovaries	No. of COCs (average/ovary)	Cleavage(%)	Compact morula (%)	Total Blastocysts (%)

**Without LF or CL**	107 (21.4)	83.64 ± 7.04^a^	45.70 ± 9.9^a^^b^	32.92 ± 5.36^b^
**At least one LF**	104 (20.8)	68.43 ± 5.18^a^	36.60 ± 5.77^b^	52.37 ± 10.04^a^
**One CL**	176 (22)	73.95 ± 7.83^a^	48.20 ± 1.51^a^	54.85 ± 9.84^a^


Data are shown as means ± SE calculated from each replicate. LF and CL represent large follicles and corpus luteum, respectively. Different letters within the same column show significant differences among the groups (p≤0.05).

### Oocyte recovery

As shown, the highest average oocytes collected per ovary were related to the CL (22), WLCF (21.4) and LF groups (20.8), respectively.

### Cleavage

There were no significant differences between cleavage rates of the different groups [WLCF group (83.6%), CL group (73.95%) and LF group (68.4%)].

### Morula

The rate of embryos in the compact morula stage for the CL group was 48.2% which was significantly higher than the related rate of the LF group (36.60%), but not significantly higher than that of the WLCF group (45.7%) (p<0.05).

### Blastocyst

Comparison between the percentages of blastocysts that developed in each group showed the highest blastocyst rate in the CL group (54.6%) which was significantly superior to the ST group (32.9%) and non-significantly higher than the LF group (52.4%). There was no significant difference between blastocyst rates in the CL and LF groups (p≤0.05).

## Discussion

Many of the oocytes collected from slaughterhouse ovaries fail to develop into viable embryos after *in vitro* maturation/fertilization processes and only 30-40% reach the blastocyst stage (14). To increase the numbers of oocytes with good developmental competence it is necessary to know the relation between cyclic state of the ovary with their developmental capacity with consideration of the LF and CL (2, 10).

The first point highlighted in this study was that the cyclic status of the ovary, in terms of the presence of CL or LF, had no significant effect on the cleavage rate of the retrieved oocytes (Table 1).

In this regards, Rizos et al. (15) have suggested that the potential of inseminated oocytes for the first mitotic divisions is more a reflection of its competency acquired during follicular development than the effect of *in vitro* culturing. A through literature review has also indicated that the cleavage rate of the embryos which develop under different culture conditions are most likely not significantly different, and hence, one may interpret it as actually the intrinsic quality of the oocyte itself that is the key factor in determining the first zygotic division (15-17). Therefore, our results can be considered as a step toward these observations and suggests that the cyclic status of the ovary at the time of oocyte collection does not compromise the quality of the developing oocytes to drive the first embryonic divisions.

During days three to four of bovine *in vitro* embryo development, which is concurrent with the critical window of maternal-zygotic transition or developmental block (18), the potential reserve of maternally inherited mRNA crucially determines the competency of embryos to progress beyond this stage. Accordingly, the results of this study have indicated that embryos derived from ovaries which contained a LF had significantly lower competency to reach the compact morula stage compared to embryos derived from ovaries that contained a CL.

The ability of *in vitro* matured and fertilized oocytes to develop to blastocysts has been considered among the important factors determining both the quality of oocytes and the efficiency of *in vitro* culture conditions (17). In this regards, the result of this study indicated that despite minor effects observed during cleavage and the compact morula stage, the cyclic status of the ovary had a profound effect on the capability of the oocytes to develop to the blastocyst stage. Importantly, it was found that neither a functional CL nor LF compromised development of blastocysts compared to oocytes derived from ovaries with no sign of either a functional CL or LF. Therefore, it can be stated that oocytes which have been influenced with the known/unknown effects of either CL or LF have greater developmental competence rather than oocytes that develop in the absence of these factors.

## Conclusion

The results of this study indicated that regardless of the culture condition, while cleavage rate is a good measure for developmental competence, the intrinsic quality of the oocyte finally determines in vitro developmental competence up to the blastocyst stage. Indeed ovary morphology is a non-invasive criteria to access developmental competence of those oocytes extracted from slaughterhouse ovaries.

## References

[1] Fukuda Y, Ichikawa M, Naito K, Toyoda Y (1990). Birth of normal calves resulting from bovine oocytes matured, fertilized, and cultured with cumulus cells in vitro up to the blastocyst stage. Biol Reprod.

[2] Gonzalez-Bulnes A, Berlinguer F, Cocero MJ, Garcia- Garcia RM, Leoni G, Naitana S (2005). Induction of the presence of corpus luteum during superovulatory treatments enhances in vivo and in vitro blastocysts output in sheep. Theriogenology.

[3] Ross P, Rodriguez R, Iager A, Beyhan Z, Wang K, Ragina N (2009). Activation of bovine somatic cell nuclear transfer embryos by PLCZ cRNA injection. Reproduction.

[4] Cibelli J (2007). Developmental biology.A decade of cloning mystique. Science.

[5] Hosseini SM, Moulavi F, Hajian M, Abedi P, Forouzanfar M, Ostad Hosseini S (2008). Highly efficient in vitro production of bovine blastocyst in cell-free sequential oviductal fluid vs.TCM199 Vero cell co-culture system. International Journal of Fertility and Sterility (IJFS).

[6] Ireland JJ, Roche JF (1983). Development of nonovulatory antral follicles in heifers: changes in steroids in follicular fluid and receptors for gonadotropins. Endocrinology.

[7] Ireland JJ, Roche JF (1983). Growth and differentiation of large antral follicles after spontaneous luteolysis in heifers: changes in concentration of hormones in follicular fluid and specific binding of gonadotropins to follicles. J Anim Sci.

[8] Murphy BD (2000). Models of luteinization. Biol Reprod.

[9] Lucy MC, Savio JD, Badinga L, De La Sota RL, Thatcher WW (1992). Factors that affect ovarian follicular dynamics in cattle. J Anim Sci.

[10] Vassena R, Adamsb GP, Mapletoft RJ, Pierson RA, Singh J (2003). Ultrasound image characteristics of ovarian follicles in relation to oocyte competence and follicular status in cattle. Animal Reproduction Science.

[11] Moulavi F, Hosseini SM, Ashtiani SK, Shahverdi A, Nasr Esfahani MH (2006). Can Vero cell co-culture improve in-vitro maturation of bovine oocytes?. Reprod Biomed Online.

[12] Shirazi A, Shams-Esfandabadi N, Hosseini S (2005). M.A comparison of two recovery methods of ovine oocytes for in vitro maturation. Small Rumin Res.

[13] Hosseini SM, Forouzanfar M, Hajian M, Asgari V, Abedi P, Hosseini L (2009). Antioxidant supplementation of culture medium during embryo development and/or after vitrification- warming; which is the most important?. J Assist Reprod Genet.

[14] Lonergan P (2007). State-of-the-art embryo technologies in cattle. Soc Reprod Fertil.

[15] Rizos D, Ward F, Duffy P, Boland MP, Lonergan P (2002). Consequences of bovine oocyte maturation, fertilization or early embryo development in vitro versus in vivo: implications for blastocyst yield and blastocyst quality. Mol Reprod Dev.

[16] Vassena R, Mapletoft RJ, Allodi S, Singh J, Adams GP (2003). Morphology and developmental competence of bovine oocytes relative to follicular status. Theriogenology.

[17] Lonergan P, Rizos D, Gutierrez-Adan A, Fair T, Boland MP (2003). Oocyte and embryo quality: Effect of origin, culture conditions and gene expression patterns. Reprod Dmest Anim.

[18] Barnes FL, Eyestone WH (1990). Early cleavage and the maternal zygotic transition in bovine embryos. Theriogenology.

